# Supplementary material for: Prediction of fluid intelligence from T1-w MRI images: A precise two-step deep learning framework
Source: PLoS One. 2022 Aug 2;17(8):e0268707. doi: 10.1371/journal.pone.0268707 (PMC9345352; doi:10.1371/journal.pone.0268707)
Supplement: S1 File — (DOCX) [file pone.0268707.s001.docx]

**Fluid Intelligence Score Measurement**

Fluid intelligence is the main component that determines general intelligence and refers to the ability to reason, analyze, and solve problems. Determining the neural mechanisms underlying fluid intelligence has important implications for understanding cognitive development and establishing effective interventions to improve adolescent health. The fluid intelligence scores recorded by the ABCD study were measured via the NIH Toolbox Neurocognition battery.

The NIH Toolbox Cognition Battery, recommended for ages 9-10, consists of tests of multiple constructs. It yields individual measure scores and the summary score of fluid cognition composite score (includes Attention & Executive Function, Working Memory, Language and Processing Speed). The test Construct details is presented in Table 1.

**Table 1. NIH Toolbox Cognition Battery**

| Construct | Measure | Description | Test Time (minutes) | Materials Required |
| --- | --- | --- | --- | --- |
| Attention & Executive Function | Flanker Inhibitory Control and Attention Test | The allocation of one’s limited capacities to deal with an abundance of environmental stimulation. | 3 | √ |
| Working Memory | List Sorting Working Memory Test | The ability to store information until the amount of information to be stored exceeds one’s capacity to hold that information. | 7 | √ |
| Language | Picture Vocabulary | Measures receptive vocabulary administered in a computer-adaptive test (CAT) format. Respondents select the picture that most closely matches the meaning of the word. | 4 |  |
| Language | Oral Reading Recognition | Measures reading decoding skill and crystallized abilities. Participant is asked to read and pronounce letters and words as accurately as possible. | 3 | √ |
| Processing Speed | Pattern Comparison Processing Speed | Assesses the amount of information that can be processed within a certain unit of time. Items are simple so as to purely measure processing speed. | 3 |  |

**Note:** **Time test (minutes) denotes time to complete in minutes; √ denotes additional materials or equipment needed.**
